# Supplementary material for: Thalamo-hippocampal pathway regulates incidental memory capacity in mice
Source: Nat Commun. 2022 Jul 20;13:4194. doi: 10.1038/s41467-022-31781-8 (PMC9300669; doi:10.1038/s41467-022-31781-8)
Supplement: Supplementary file 2 — Reporting Summary [file 41467_2022_31781_MOESM2_ESM.pdf]

nature portfolio

Corresponding author(s): Dr. Elvira De Leonibus
Last updated by author(s): Jun 3, 2022

Reporting Summary

Nature Portfolio wishes to improve the reproducibility of the work that we publish. This form provides structure for consistency and transparency in reporting. For further information on Nature Portfolio policies, see our Editorial Policies and the Editorial Policy Checklist.

Statistics

For all statistical analyses, confirm that the following items are present in the figure legend, table legend, main text, or Methods section.

n/a	Confirmed	
☐	☒	The exact sample size (n) for each experimental group/condition, given as a discrete number and unit of measurement
☐	☒	A statement on whether measurements were taken from distinct samples or whether the same sample was measured repeatedly
☐	☒	The statistical test(s) used AND whether they are one- or two-sided<br>*Only common tests should be described solely by name; describe more complex techniques in the Methods section.*
☒	☐	A description of all covariates tested
☐	☒	A description of any assumptions or corrections, such as tests of normality and adjustment for multiple comparisons
☐	☒	A full description of the statistical parameters including central tendency (e.g. means) or other basic estimates (e.g. regression coefficient) AND variation (e.g. standard deviation) or associated estimates of uncertainty (e.g. confidence intervals)
☐	☒	For null hypothesis testing, the test statistic (e.g. F, t, r) with confidence intervals, effect sizes, degrees of freedom and P value noted<br>*Give P values as exact values whenever suitable.*
☒	☐	For Bayesian analysis, information on the choice of priors and Markov chain Monte Carlo settings
☒	☐	For hierarchical and complex designs, identification of the appropriate level for tests and full reporting of outcomes
☐	☒	Estimates of effect sizes (e.g. Cohen's d, Pearson's r), indicating how they were calculated

Our web collection on statistics for biologists contains articles on many of the points above.

Software and code

Policy information about availability of computer code

Data collection	ANY-MAZE Version 3, Stoelting, USA; NIS-Elements C 4.20 (Nikon, Florence, Italy); Leica Application Suite X (LAS-X; Leica Microsystems GmbH, Germany).
Data analysis	Statview 5.0, Statistica 7, GraphPad Prism 8 and G*power 3.1, FIJI (ImageJ), QuPath (Bankhead, P. et al. 2017).

For manuscripts utilizing custom algorithms or software that are central to the research but not yet described in published literature, software must be made available to editors and reviewers. We strongly encourage code deposition in a community repository (e.g. GitHub). See the Nature Portfolio guidelines for submitting code & software for further information.

Data

Policy information about availability of data

All manuscripts must include a data availability statement. This statement should provide the following information, where applicable:

- Accession codes, unique identifiers, or web links for publicly available datasets
- A description of any restrictions on data availability
- For clinical datasets or third party data, please ensure that the statement adheres to our policy

All data is available upon reasonable request to the corresponding author. Source data underlying the main and supplementary figures are provided as a Source Data file.

Field-specific reporting

Please select the one below that is the best fit for your research. If you are not sure, read the appropriate sections before making your selection.

- [x] Life sciences
- [ ] Behavioural & social sciences
- [ ] Ecological, evolutionary & environmental sciences

For a reference copy of the document with all sections, see nature.com/documents/nr-reporting-summary-flat.pdf

Life sciences study design

All studies must disclose on these points even when the disclosure is negative.

Sample size	The number of mice per group was calculated a priori with power analysis using G*Power 3.1 software with $\alpha = 0.05$ and power $(1 - \beta) = 0.80$.
Data exclusions	Significant outliers were calculated at the alpha level 0.05, through the online free software Outlier Calculator by GraphPad (available at https://www.graphpad.com/quickcalcs/Grubbs1.cfm), which employs Grubbs' test to define a significant outlier.
Replication	The reproducibility of the experimental findings was verified by replicating the same results using different cohorts of animals in different time for at least two or more times including all the experimental conditions each time.
Randomization	Mice were allocated into experimental groups in random order.
Blinding	The investigators were blinded to experimental condition.

Reporting for specific materials, systems and methods

We require information from authors about some types of materials, experimental systems and methods used in many studies. Here, indicate whether each material, system or method listed is relevant to your study. If you are not sure if a list item applies to your research, read the appropriate section before selecting a response.

Materials & experimental systems

n/a	Involved in the study	
[ ]	[x]	Antibodies
[x]	[ ]	Eukaryotic cell lines
[x]	[ ]	Palaeontology and archaeology
[ ]	[x]	Animals and other organisms
[x]	[ ]	Human research participants
[x]	[ ]	Clinical data
[x]	[ ]	Dual use research of concern

Methods

n/a	Involved in the study	
[x]	[ ]	ChIP-seq
[x]	[ ]	Flow cytometry
[x]	[ ]	MRI-based neuroimaging

Antibodies

Antibodies used	The antibodies used in this study were: anti-c-Fos (1:400, sc-52, Santa Cruz Biotechnology); anti-HA tag (1:500, #3724; Cell Signaling); anti-β-actin (1:5000, MAB1501, Millipore), anti-glutamate receptor 1 (AMPA subtype) phosphoSer 845 antibody (1:500, ab76321, Abcam); anti-glutamate receptor 1 (AMPA subtype) antibody (1:500, ab31232, Abcam); anti-c-Fos (1:1000, 226 017, Synaptic System); anti-GAD67 (1:700, MAB5406, Merck Millipore); anti-p-EIF2α (Ser51) (1:200, 3597, Cell Signaling); anti-EIF2α (1:800, 5324, Cell Signaling); anti-NeuN (1:500, ABN90, Merck Millipore); goat-anti-rabbit (1:300, Alexa Fluor 647, AP187SA6, Merck Millipore); goat anti-rat (1:400, Alexa-Fluor® 568, ab175476, abcam); goat anti-mouse (1:400, Alexa-Fluor® 488, AP124JA4, Merck Millipore); goat anti-guinea pig (1:300, Alexa-Fluor® 568, ab175714, Abcam).
Validation	We used antibodies already validated by previous studies (De Risi, Torromino et al., Aging, 2020; Torromino et al., Nature Communications, 2019) or by manufacturer for western blot or immunofluorescence. Moreover, control of specificity of immunolabeling were performed by omission of primary antibodies.

Animals and other organisms

Policy information about studies involving animals; ARRIVE guidelines recommended for reporting animal research

Laboratory animals	Adult (3 months old) male and female CD1 outbred mice were used and data are reported disaggregated for sex in all the manuscript.
Wild animals	We did not use wild animals.
Field-collected samples	No field collected samples were used in this study.

Ethics oversight	Animal studies were conducted in accordance with the guidelines and policies of the European Communities Council and were approved by the Italian Ministry of Health (authorization n° 446/2015-PR and 781/2019-PR).

Note that full information on the approval of the study protocol must also be provided in the manuscript.
